# The species, distribution, resistance of donor-derived pathogens and their impact on solid organ transplant recipients

**DOI:** 10.3389/fimmu.2026.1777244

**Published:** 2026-03-06

**Authors:** Yan-Man Zhou, Xian-Quan Cui, Peng Zhao, Zhi-Guo Peng, Ning Guo, Huai-Bin Sun, Sheng-Li Liu

**Affiliations:** 1 Department of Nephrology, Shandong Provincial Hospital Affiliated to Shandong First Medical University, Jinan, Shandong, China; 2 Department of Organ Transplantation, Qilu Hospital of Shandong University, Jinan, Shandong, China

**Keywords:** donor-derived infection, metagenomic next-generation sequencing, multidrug-resistant organism, preservation fluid, solid organ transplantation

## Abstract

**Background:**

Donor-derived infections (DDIs) have become a significant cause of infection in organ transplant recipients. Elaborating on the species, distribution, and resistance of donor-derived pathogens (DDPs) holds important implications.

**Methods:**

A retrospective cohort study included 302 deceased donors and their corresponding 464 kidney transplant recipients and 175 liver transplant recipients. We detected DDPs in preservation fluid (PF) using both conventional culture and mNGS, and subsequently analyzed the incidence of DDIs after transplantation.

**Results:**

89.4% (270/302) of donors had positive cultures. Predominant multidrug-resistant organism included HLAR-*Enterococcus*, CRAB, CRKP, CRPA, MRS and ESBL-*Escherichia coli*. Compared with conventional culture, mNGS exhibited superior sensitivity for detecting bacteria and fungus in PF, with shorter turnaround time (p < 0.001). The incidences of DDIs in kidney and liver transplant recipients were 16.6% (77/464) and 19.4% (34/175) respectively. The recipients with DDIs were associated with elevated serum creatinine or total bilirubin levels, increased infection events, higher risks of graft loss, elevated mortality, and longer length of hospital stay (p < 0.05).

**Conclusions:**

Multidrug-resistant organism are prevalent in deceased donors, with PF contamination primarily originating from donors. Integration of mNGS into donor screening protocols enables timely antimicrobial intervention, potentially improving transplant outcomes.

## Introduction

Nowadays, solid organ transplantation is the optimal treatment for end-stage organ failure, yet recipients face elevated risks of healthcare-associated infections due to sustained immunosuppression (1–4). At present, the reliance on deceased donors, along with the expanded use of marginal donors to solve organ shortage, has increased the incidence of donor-derived infections (DDIs) (5, 6). Potential donors in the intensive care units (ICU) are frequently colonized with multidrug-resistant organisms via invasive devices and wounds, which may transmit to recipients through the graft and lead to severe complications such as surgical site infection, poor wound healing, graft dysfunction, sepsis, and even death (7, 8).

Preservation fluid (PF) is essential for maintaining organ viability, can also serve as a medium for the proliferation and transmission of donor-derived pathogens (DDPs) (9, 10). Although PF culture is widely adopted in transplant protocols, its utility is limited by prolonged turnaround time and suboptimal sensitivity (11, 12). Furthermore, variability in clinical practices across different centers contributes to the inconsistent rates of DDIs reported in the literature, which range from 0.18% to 20%, with *Enterococcus* and coagulase-negative *Staphylococcus* being commonly isolated (6, 12).

To overcome these challenges, our center has adopted an integrated diagnostic strategy combining conventional PF culture with metagenomic next-generation sequencing (mNGS). Depends on our high-volume transplantation platform, this study aims to comprehensively characterize the species, distribution, antimicrobial resistance profiles, and clinical impact of DDPs on recipients.

## Methods

### Study design and cohort selection criteria

This retrospective study included 495 kidney transplant recipients and 202 liver transplant recipients at Qilu Hospital of Shandong University between January 2019 and June 2025. Through tracing the donors corresponding to these recipients, 302 deceased donors with complete information were identified. All donors were voluntary and had been determined to be cardiac dead, brain dead, or both at the time of donation. These organs were allocated via the China Organ Transplant Response System. After excluding cases with perioperative cardiovascular events, cerebrovascular events, gastrointestinal hemorrhage, transplant-associated vascular complications, or pulmonary infections, we ultimately included 464 kidney transplant recipients and 175 liver transplant recipients for analysis. Furthermore, the study protocol was approved by the local ethics committee and conducted in accordance with the Declaration of Helsinki (KYLL-2025SL-015). All data involved in this study were obtained with informed consent from the recipients and the legal representatives of donors.

### Definition of DDPs and DDIs

All donors routinely underwent two or more sets of blood cultures, urine cultures, and respiratory secretion cultures (including sputum and bronchoalveolar lavage fluid) during their ICU management. Targeted anti-infection therapy was administered based on antimicrobial susceptibility testing results. The final pre-donation culture results served as the basis for assessing whether active infections persisted in donors.

Following en bloc procurement of donor liver and kidneys, organs were preserved in cold hypertonic citrate adenine solution (HC-A, Shanghai Blood Transfusion Technology Co., Ltd, China). Prior to organ separation, four samples (10 ml each) of PF were collected under sterile conditions. Two samples underwent conventional culture, while the remaining two samples were subjected to mNGS. Samples with similar results within the same testing modality were deemed valid. When culture or mNGS of PF demonstrated concordance in pathogen species and antimicrobial resistance profiles with the donor’s blood, urine, or respiratory secretion cultures, the pathogens were classified as DDPs.

For liver and kidney recipients, two or more sets of drainage fluid were obtained to culture within the first 3–5 days after transplantation. Based on the strength of available evidence, we have introduced a broad definition with specific inclusion and exclusion criteria for DDIs. We included infected recipients from whom pathogens were cultured from drainage fluid and demonstrated concordance at species level and antimicrobial resistance profiles with pathogens detected either by culture or mNGS from the corresponding PF of donors. Besides, these pathogens were also consistent with those identified in the donor’s blood, urine, or respiratory secretion cultures, indicating their presence in the donor at the time of donation. Furthermore, the recipient’s infection occurred during the early post-transplant hospitalization period, with no alternative plausible source identified. Recipients were excluded if there was evidence that the infection originated from exogenous contamination events, or unrelated to this surgery. Meanwhile, we documented infectious events in all recipients, including bacteremia, urinary tract infection (UTI), graft site infection, abdominal infection, biliary fistula and wound infection. All recorded infectious events occurred during the hospitalization period immediately after transplantation.

### Microbiological culture and antimicrobial susceptibility testing

After centrifugation at 3000 × g for 10 minutes, the sediment of cultures were inoculated onto specific culture media selected based on the type of pathogens, followed by regular monitoring of microbial growth. Pathogen identification was initially performed by examining colony morphology and gram staining results, with definitive identification achieved using an automated microbial identification system and mass spectrometry. Antimicrobial susceptibility testing (AST) was subsequently conducted via the broth microdilution method, and all susceptibility results were interpreted in accordance with the standards established by the Clinical and Laboratory Standards Institute (13).

### mNGS detection

mNGS detection was performed by WillingMed Technology (Beijing, China). Firstly, red blood cells and other impurities were depleted from PF of donors to enrich potentially pathogens. Then microbial DNA or RNA was extracted, followed by removal of host nucleic acids. Subsequently, the extracted nucleic acids were subjected to fragmentation and adapter ligation to construct a library. The library was sequenced on the illumina platform to generate nucleic acid sequence data. The data were filtered and matched against the China Pneumonia Research Network Database to identify pathogens. A sample was generally considered positive when ≥20 sequence reads for bacteria and fungus, or ≥3 sequence reads for virus.

### Immunosuppressive regimens and antimicrobial prophylaxis protocols

The immunosuppressive regimen followed international standards, including induction therapy with anti-thymocyte globulin (ATG) combined with high-dose corticosteroids, followed by tapered maintenance corticosteroids, tacrolimus, and mycophenolate mofetil after transplantation (14). For recipients of grafts from donors without evidence of infection, the antimicrobial prophylaxis protocol consisted of carbapenems combined with caspofungin for 7 days. When DDPs were identified in PF culture or mNGS, the prophylactic regimen was tailored according to the pathogen species, resistance profiles, and AST results (**Supplementary Table 1**).

### Statistical analysis

Continuous variables were presented as mean ± SD or median. Categorical variables were summarized as frequencies and percentages. Statistical analyses were performed using descriptive statistics, Mann-Whitney and chi-square tests, with p-value < 0.05 considered statistically significant. Binary variables with p-value < 0.05 were incorporated into multivariate analysis. Results are reported as odds ratios (OR) with 95% confidence intervals (95% CI). All analyses were performed using statistical software (SPSS 18.0).

## Results

### The general characteristics and infection status of deceased donors

Among the 302 deceased donors, 89.4% (270/302) tested positive for pathogens in blood, respiratory secretion, or urine. No differences were observed between the infected and non-infected donor groups regarding age, gender, cause of death, pre-donation total bilirubin (TBIL) or serum creatinine (SCR) levels. However, significant differences were found in the endotracheal intubation days and length of stay in hospital. Subsequent multivariate analysis identified only the endotracheal intubation days and length of stay in hospital as independent factors associated with donor infection (OR 0.904, 95% CI 0.885-0.924; OR 0.913, 95% CI 0.895-0.930. **Supplementary Table 2**).

For infected donors, conventional culture revealed positive rates of 14.2% (43/302) in blood, 84.4% (255/302) in respiratory secretion, and 20.5% (62/302) in urine. Regardless of the types of body fluid, the predominant bacterial species were *Enterococcus*, *Klebsiella pneumoniae*, *Acinetobacter baumannii*, *Pseudomonas aeruginosa*, *Staphylococcus*, *Escherichia coli*, and *Enterobacter cloacae*, with the majority being multidrug-resistant strains. The resistance profiles were characterized by high-level aminoglycoside resistance (HLAR) in *Enterococcus*, carbapenem resistance in *Klebsiella pneumoniae*, *Acinetobacter baumannii*, *Pseudomonas aeruginosa* (CRKP, CRAB, CRPA), methicillin resistance in *Staphylococcus* (MRS), and extended-spectrum β-lactamase (ESBL) production in *Escherichia coli*. The species of fungus were primarily *Candida*, *Aspergillus*, without significant resistance (**Table 1**).

**Table 1:** The status of cultured pathogens in deceased donors.

Pathogens	Blood (n=43, 14.2%)		Respiratory secretion (n=255, 84.4%)		Urine (n=62, 20.5%)	
	Non-DR (n=13, 30.3%)	DR (n=30, 69.7%)	Non-DR (n=83, 32.6%)	DR (n=172, 67.4%)	Non-DR (n=30, 48.4%)	DR (n=32, 51.6%)
Gram-positive cocci						
*Staphylococcus*	3	12	3	13	1	5
*Enterococcus*	2	7	4	8	2	7
*Streptococcus*	0	0	14	0	0	0
Gram-negative bacilli						
*Acinetobacter baumannii*	1	2	6	56	2	3
*Pseudomonas aeruginosa*	0	2	4	39	1	3
*Klebsiella pneumoniae*	0	3	3	48	1	4
*Escherichia coli*	1	4	2	6	5	10
*Enterobacter cloacae*	1	0	4	0	2	0
*Haemophilus influenzae*	0	0	5	0	0	0
*Burkholderia cepacia*	0	0	3	0	0	0
*SMA*	0	0	5	0	0	0
*Others*	2	0	9	2	0	0
Fungus						
*Candida*	2	0	10	0	14	0
*Aspergillus*	1	0	11	0	2	0

DR, Drug-resistant; Non-DR, Non-drug-resistant; *SMA, Stenotrophomonas maltophilia.*

### Pathogen profiles in PF and comparison of detection methods

In PF samples of transplanted organs, results from both mNGS and conventional culture demonstrated that the species of bacteria and fungus, along with their resistance profiles, closely resembled those found in the donor’s body fluid culture. As virus cannot be routinely cultured, mNGS alone identified the most prevalent virus species as polyomavirus, herpesvirus, and anellovirus (**Table 2**).

**Table 2:** The species and resistance profiles of pathogens detected by conventional culture and mNGS in PF.

Pathogens	Culture			mNGS			P-value
	Non-DR	DR	Percentage	Number of cases	Sequences, median	Percentage	
Bacteria	12	45	18.9% (57/302)	223		73.8% (223/302)	**0.000**
*Staphylococcus*	1	2		42	20-80982, 240		
*Enterococcus*	3	12		125	21-773280, 276		
*Streptococcus*	0	0		23	21-467, 34		
*Acinetobacter baumannii*	1	9		59	20-702048, 746		
*Pseudomonas aeruginosa*	0	7		52	20-12687, 240		
*Klebsiella pneumoniae*	1	12		70	20-345019, 193		
*Escherichia coli*	2	3		48	22-46567, 95		
*Enterobacter cloacae*	2	0		28	22-29196, 234		
*Haemophilus influenzae*	0	0		11	21-896, 39		
*Burkholderia cepacia*	0	0		9	23-3463, 67		
*SMA*	1	0		16	21-97293, 40		
*Serratia marcescens*	1	0		14	20-14452, 133		
*Others*	0	0		27	21-3646, 53		
Fungus	2	0	0.6% (2/302)	71		23.5% (71/302)	**0.000**
*Candida*	1	0		42	21-58223, 118		
*Aspergillus*	1	0		35	20-805, 58		
*Saccharomycetes*	0	0		24	21-754, 45		
*Rhizopus*	0	0		4	21-47, 32		
Virus	/	/	/	99		32.8% (99/302)	
*Polyomavirus*	/	/		70	3-5018, 23		
*Herpesvirus*	/	/		49	4-609, 12		
*Anellovirus*	/	/		38	3-427, 7		
*Others*	/	/		17	3-85, 19		

mNGS, metagenomic next-generation sequencing; PF, Preservation fluid; DR, Drug-resistant; Non-DR, Non-drug-resistant; *SMA, Stenotrophomonas maltophilia.*Bold values indicate statistical significance (p < 0.05).

For both bacteria and fungus, mNGS demonstrated significantly higher positive detection rates in pathogens of PF compared to conventional culture (73.8% (223/302) versus 18.9% (57/302), p < 0.001; 23.5% (71/302) versus 0.6% (2/302), p < 0.001. **Table 2**), with a considerably shorter turnaround time (24.0 ± 0.00 versus 84.0 ± 4.17, p < 0.001, **Table 3**). These thereby allow us for timely adjustment of antimicrobial therapy in recipients. Furthermore, mNGS outperformed conventional culture in determining the DDPs, with detection sensitivity being highest in blood (93.0% (40/43) versus 46.5% (20/43), p < 0.001), followed by urine and respiratory secretions of donors (72.6% (45/62) versus 17.7% (11/62), p < 0.001; 58.8% (150/255) versus 9.8% (25/255), p < 0.001. **Table 3**).

**Table 3:** The superior sensitivity and speed of mNGS for DDPs detection.

Sensitivity	Culture	mNGS	P-value
Consuming time (hours)	84.0 ± 4.17	24.0 ± 0.00	**0.000**
Blood, n (%)	46.5% (20/43)	93.0% (40/43)	**0.000**
Respiratory secretion, n (%)	9.8% (25/255)	58.8% (150/255)	**0.000**
Urine, n (%)	17.7% (11/62)	72.6% (45/62)	**0.000**

mNGS, metagenomic next-generation sequencing; DDPs, Donor-derived pathogens.Bold values indicate statistical significance (p < 0.05).

### The characteristics of DDPs in recipient drainage fluid and their correlation with PF

Among 464 kidney transplant recipients, 88 had pathogen-positive drainage fluid cultures. Based on the previous definition and excluding non-DDIs events (11 recipients), the incidence of DDIs was 16.6% (77/464). The predominant bacterial species were HLAR-*Enterococcus*, CRAB, CRKP, CRPA, and ESBL-producing *Escherichia coli*. Besides, the correlation analysis between drainage fluid and PF revealed that mNGS demonstrated significantly higher sensitivity than conventional culture (86.7% (72/83) versus 53.0% (44/83), p < 0.001. **Table 4**). The donor-derived fungus were primarily *Candida* and *Aspergillus*, with significant difference between mNGS and conventional culture (100% (5/5) versus 40.0% (2/5), p = 0.038. **Table 4**).

**Table 4:** The basic characteristics of DDPs in recipient drainage fluid and their correlation with PF.

Pathogens	KT					LT				
	Non-DR	DR	PF-culture (%)	PF-mNGS (%)	P-value	Non-DR	DR	PF-culture (%)	PF-mNGS (%)	P-value
Bacteria	13	70	53.0% (44/83)	86.7% (72/83)	**0.000**	6	34	47.5% (19/40)	80.0% (32/40)	**0.002**
*Staphylococcus*	1	3	50.0% (2/4)	75.0% (3/4)		1	1	50.0% (1/2)	50.0% (1/2)	
*Enterococcus*	3	20	43.5% (10/23)	86.9% (20/23)		2	11	46.2% (6/13)	69.2% (9/13)	
*Acinetobacter baumannii*	0	16	50.0% (8/16)	81.3% (13/16)		0	7	42.9% (3/7)	85.7% (6/7)	
*Pseudomonas aeruginosa*	0	10	70.0% (7/10)	90.0% (9/10)		0	5	60.0% (3/5)	100% (5/5)	
*Klebsiella pneumoniae*	1	13	64.3% (9/14)	85.7% (12/14)		1	7	50.0% (4/8)	87.5% (7/8)	
*Escherichia coli*	2	8	40.0% (4/10)	90.0% (9/10)		1	3	50.0% (2/4)	75.0% (3/4)	
*Enterobacter cloacae*	3	0	66.7% (2/3)	100% (3/3)		1	0	100% (1/1)	100% (1/1)	
*SMA*	1	0	100% (1/1)	100% (1/1)		0	0	/	/	
*Serratia marcescens*	2	0	50.0% (1/2)	100% (2/2)		0	0	/	/	
Fungus	5	0	40.0% (2/5)	100% (5/5)	**0.038**	2	0	50.0% (1/2)	100% (2/2)	0.248
*Candida*	3	0	50.0% (1/3)	100% (3/3)		1	0	100% (1/1)	100% (1/1)	
*Aspergillus*	2	0	0.0% (1/2)	100% (2/2)		1	0	0.0% (0/1)	100% (1/1)	
Virus	/	/	/	/	/	/	/	/	/	/

DDPs, Donor-derived pathogens; PF, Preservation fluid; DR, Drug-resistant; Non-DR, Non-drug-resistant; KT, Kidney transplantation; LT, Liver transplantation; mNGS, metagenomic next-generation sequencing; *SMA, Stenotrophomonas maltophili.*Bold values indicate statistical significance (p < 0.05).

For 175 liver transplant recipients, 42 had pathogen-positive drainage fluid cultures. After excluding non-DDIs events (8 recipients), the incidence of DDIs was 19.4% (34/175). The species and resistance profiles of bacteria were similar to those observed in kidney transplant recipients. The correlation analysis between drainage fluid and PF revealed that mNGS demonstrated higher positive detection rate than conventional culture (80.0% (32/40) versus 47.5% (19/40), p = 0.002. **Table 4**). The donor-derived fungus were also primarily *Candida* and *Aspergillus*, and due to the limited number of infected recipients, there was no significant difference between mNGS and conventional culture (100% (2/2) versus 50.0% (1/2), p = 0.248. **Table 4**).

### Clinical outcomes associated with DDIs in kidney transplant recipients

The kidney transplant recipients with DDIs demonstrated no significant differences from the control group in terms of age, gender, types of primary disease, dialysis time, numbers of HLA mismatch, cold ischemia time, incidence of delayed graft function (DGF), and pretransplant SCR levels (p > 0.05, **Table 5**). However, once obtained DDIs, these recipients may experience deterioration in allograft function, characterized by persistently elevated SCR levels, substantially increased infection events, higher risks of graft loss, elevated mortality, and longer perioperative length of hospital stay (174.8 ± 12.67 versus 137.0 ± 4.74; 20 (30.0%) versus 13 (3.4%), 42 (54.5%) versus 43 (11.1%), 36 (46.7%) versus 33 (8.5%), 15 (19.5%) versus 7 (1.8%); 17 (22.0%) versus 8 (2.1%); 11 (14.3%) versus 6 (1.6%); 22.5 ± 1.30 versus 16.7 ± 0.30. p < 0.05, **Table 5**).

**Table 5:** Impact of DDIs on kidney transplant recipients.

Characteristics	Control (n=387)	DDIs (n=77)	P-value
Age (years)	43.4 ± 0.70	42.8 ± 1.45	0.941
Gender, male, n (%)	218 (56.3%)	39 (50.6%)	0.360
Cause of ESRD, n (%)			0.606
Glomerulonephritis	173 (44.7%)	42 (54.5%)	
Diabetes	94 (24.3%)	19 (24.7%)	
Hypertension	74 (19.1%)	10 (13.0%)	
Polycystic kidney	21 (5.4%)	3 (3.9%)	
Others	25 (6.5%)	3 (3.9%)	
Dialysis time (months)	28.0 ± 1.06	32.3 ± 2.37	0.350
HLA-MM			0.257
0-3	281 (72.6%)	51 (66.2%)	
4-6	106 (27.4%)	26 (33.8%)	
Cold ischemia time (hours)	5.2 ± 1.67	5.4 ± 1.89	0.457
DGF, n (%)	61 (15.8%)	18 (23.4%)	0.104
SCR (umol/L)			
Before KT	872.5 ± 17.67	818.4 ± 32.10	0.172
After KT	137.0 ± 4.74	174.8 ± 12.67	**0.001**
Infectious events, n (%)			0.008
Graft site infection	13 (3.4%)	20 (30.0%)	
Urinary tract infection	43 (11.1%)	42 (54.5%)	
Wound infection	33 (8.5%)	36 (46.7%)	
Bacteremia	7 (1.8%)	15 (19.5%)	
Graft loss, n (%)	8 (2.1%)	17 (22.0%)	**0.000**
Dead, n (%)	6 (1.6%)	11 (14.3%)	**0.000**
Length of stay (days)	16.7 ± 0.30	22.5 ± 1.30	**0.000**

DDIs, Donor-derived infections; ESRD, End stage renal disease; HLA-MM, HLA mismatch; DGF, Delayed graft function; SCR, Serum creatinine; KT, Kidney transplantation.Bold values indicate statistical significance (p < 0.05).

### Clinical outcomes associated with DDIs in liver transplant recipients

Compared to the control group, the liver transplant recipients with DDIs showed no differences in age, gender, types of primary disease, pretransplant TBIL levels and cold ischemia time (p > 0.05, **Table 6**). However, in contrast, the latter demonstrated significantly higher posttransplant TBIL levels, increased incidence of infection events, elevated mortality, and longer perioperative length of hospital stay (42.2 ± 6.64 versus 26.8 ± 1.58; 22 (64.8%) versus 32 (22.7%), 12 (35.3%) versus 22 (15.6%), 17 (50.0%) versus 28 (19.9%), 9 (26.5%) versus 11 (7.8%); 11 (32.4%) versus 15 (10.6%); 32.3 ± 2.34 versus 24.8 ± 0.85. p < 0.05, **Table 6**).

**Table 6:** Impact of DDIs on liver transplant recipients.

Characteristics	Control (n=141)	DDIs (n=34)	P-value
Age (years)	48.5 ± 0.98	45.2 ± 2.15	0.144
Gender, male, n (%)	94 (66.7%)	24 (70.6%)	0.661
Cause of liver failure, n (%)			0.855
Viral cirrhosis	64 (45.3%)	16 (47.1%)	
Alcoholic cirrhosis	24 (17.0%)	7 (20.6%)	
Autoimmune cirrhosis	18 (12.8%)	3 (8.8%)	
Tumor	22 (15.6%)	5 (14.7%)	
Others	13 (9.3%)	3 (8.8%)	
TBIL (umol/L)			
Before LT	104.8 ± 13.08	82.2 ± 15.69	0.396
After LT	26.8 ± 1.58	42.2 ± 6.64	**0.000**
Cold ischemia time (hours)	7.3 ± 1.42	7.7 ± 1.12	0.271
Infectious events, n (%)			0.022
Abdominal infection	32 (22.7%)	22 (64.8%)	
Biliary fistula	22 (15.6%)	12 (35.3%)	
Wound infection	28 (19.9%)	17 (50.0%)	
Bacteremia	11 (7.8%)	9 (26.5%)	
Dead, n (%)	15 (10.6%)	11 (32.4%)	**0.001**
Length of stay (days)	24.8 ± 0.85	32.3 ± 2.34	**0.000**

DDIs, Donor-derived infections; TBIL, Total bilirubin; LT, Liver transplantation.Bold values indicate statistical significance (p < 0.05).

## Discussion

With the increasing number of solid organ transplantation, DDIs for recipients have become a major clinical focus due to their high pathogenicity, elevated mortality, and therapeutic difficulties (15, 16). In this study, bacteria detected in blood, respiratory secretion, and urine of deceased donors were predominant multidrug-resistant organism, and fungus exhibited minimal drug resistance. These findings collectively reflect the characteristic infection profile of donors in our center. The infections of multidrug-resistant organism are significantly associated with factors such as the patient having suffered severe trauma, received broad-spectrum antimicrobial therapy, undergone invasive procedures, and experienced prolonged hospitalization (17). Therefore, when other factors are non-modifiable, promptly initiating the organ donation process following determination of cardiac dead, brain dead, or both in potential donors is imperative to reduce the duration of hospitalization and minimize infectious opportunities. Our center employs carbapenems and caspofungin as the baseline prophylactic regimen after transplantation, a decision based on the need to address the high prevalence of multidrug-resistant organism in the Chinese ICU environment, particularly among potential donors. During the critical window period when the mNGS or culture results of donor PF are not immediately available, the regimen aims to provide recipients with broad-spectrum coverage against the most common and life-threatening pathogens, thereby preventing early and fulminant postoperative infections.

PF is collected directly from the perfusate of the transplanted organ or cavity secretions, provides a more direct reflection of the microbial colonization status within the organ tissue (18). In this study, the species and resistance profiles of pathogens isolated from PF were largely consistent with those found in the blood, urine, and respiratory secretions of donors. This finding indicates that the pathogens of PF primarily originate from the donor rather than the external environment, a conclusion that diverges from some previous studies (19, 20). Pathogens in PF may stem directly from the body fluid or result from injury to the gastrointestinal tract or lungs of donors during the organ procurement (21). Therefore, establishing a standardized protocol for the organ procurement surgery remains essential. Furthermore, strategies such as repeated flushing of the transplanted organ prior to packaging, immersion in ex vivo-active antibiotics, or soaking in diluted povidone-iodine solution do not lose their merit as protective measures (22, 23). The positive rate of PF culture varies considerably across different regions, as reported by previous studies (11, 12, 24). These discrepancies are primarily attributable to regional differences in contextual factors such as ICU environments, broad-spectrum antibiotic usage, and duration of hospitalization. Furthermore, as a retrospective cohort study, we only enrolled already transplanted donor-recipient pairs, which may result in an inherent bias in the donor risk spectrum.

This study compared the efficacy of conventional culture versus mNGS in PF testing, demonstrating a significant advantage for mNGS. Conventional culture methods are limited by pathogen growth requirements, prolonged incubation times, and low technical sensitivity, often leading to false-negative results (25). These limitations are particularly pronounced in detecting fungus and slow-growing bacteria (26). In contrast, mNGS technology, independent of microbial cultivation, directly analyzes the genomic information of all microorganisms in a sample through high-throughput sequencing. It not only rapidly and accurately identifies pathogen species but also simultaneously detects resistance genes, providing a basis for early clinical formulation of precise anti-infective strategies (27). For organ transplantation, where time sensitivity is paramount, the rapidity and comprehensiveness of mNGS can significantly shorten the donor evaluation cycle and reduce the risk of multidrug-resistant organism transmission from donor to recipient. Of course, the clinical significance of this high positivity rate must also be interpreted with caution. mNGS detects only microbial nucleic acids, therefore, a positive result does not directly equate to the presence of viable pathogen. Besides, low sequence read counts may arise from sample contamination, background noise, or low-level colonization, and their clinical relevance requires comprehensive interpretation in conjunction with conventional culture results, antimicrobial susceptibility testing, and the patient’s clinical characteristics. Finally, it is important to emphasize that the superiority of mNGS over conventional culture in detecting pathogens from PF reflects intrinsic differences in diagnostic technology. This comparison was performed on PF samples obtained prior to any antimicrobial exposure in recipients. Therefore, the diagnostic advantage demonstrated for mNGS in PF is independent and unaffected by the recipient’s postoperative prophylactic regimen.

Both conventional culture and mNGS of PF detected pathogens predominantly consisting of drug-resistant *Enterococcus* and carbapenem-resistant *Enterobacteriaceae* (CRE). These two types of bacteria represent major clinical “superbugs” of significant concern. Drug-resistant *Enterococcus* frequently cause surgical site infections, UTIs, septicemia, and readily disseminate among immunocompromised populations (28). CRE exhibit resistance to multiple antimicrobial agents, including carbapenems, and are characterized by rapid proliferation (29). Due to limited effective treatment options, CRE infections often follow a protracted course with frequent recurrences, and associated mortality rates can reach 30%-50% (30, 31). Consequently, upon detection of these pathogens in donor organs, prompt initiation of targeted antibiotic therapy with an adequate treatment course is essential to ensure favorable recipient outcomes.

Pathogen detection in drainage fluid remains a critical step in confirming infection in recipients, as poor infection control can lead to severe adverse outcomes (32). Mechanistically, recipients require long-term immunosuppression to prevent rejection, resulting in impaired immune function and diminished capacity to clear pathogens. Meanwhile, the transplanted organ itself may harbor DDPs, serving as a source of infection that can trigger graft inflammation, dysfunction, or even failure. The finding of this study underscore the urgency of donor pathogen screening and intervention. If a donor has an uncontrolled infection, it needs to perform a comprehensive risk-benefit assessment, and even discard the organs.

In conclusion, this retrospective cohort study reveals a high prevalence of multidrug-resistant organism colonization among deceased donors at our center and demonstrates that mNGS significantly improves the pathogen detection rate in PF compared with conventional culture. These findings highlight the important clinical value of mNGS-based screening in identifying DDPs. However, when interpreting these results, several limitations must be taken into account, including the absence of genotypic confirmation in the definition of DDIs, the inability to fully exclude confounding factors such as the severity of donor infection, detailed intraoperative events, or variability in postoperative management in the statistical analysis, and the potential for selection bias in the enrollment of this study. Future prospective multicenter studies with standardized protocols are essential to validate these findings and to determine the true clinical benefit of mNGS in transplant infectious disease management. Furthermore, the postoperative use of a prophylactic regimen combining carbapenems and caspofungin may have reduced the positive culture rate in recipient drainage fluid and the incidence of DDIs, thereby affecting the objective validity of this study. Future studies employing less aggressive prophylactic strategies or standardized protocols will help clarify these issues.

## Data Availability

The original contributions presented in the study are included in the article/**Supplementary Material**. Further inquiries can be directed to the corresponding author.
