# Supplementary material for: The species, distribution, resistance of donor-derived pathogens and their impact on solid organ transplant recipients
Source: Front Immunol. 2026 Mar 6;17:1777244. doi: 10.3389/fimmu.2026.1777244 (PMC13002418; doi:10.3389/fimmu.2026.1777244)
Supplement: Supplementary file 1 [file DataSheet1.docx]

**Table S1. The anti-infection regimens for solid organ transplant recipients**

| Pathogens | Shared therapeutic agents | | | | | | |  |  | Specific therapeutic agents | | |  | Days |
| --- | --- | --- | --- | --- | --- | --- | --- | --- | --- | --- | --- | --- | --- | --- |
|  |  |  |  |  |  |  |  |  |  | KT |  | LT |  |  |
|  | Carbapenems | TGC | POLB | CAZ-AVI | GCV | CAS | VRC | ABCD |  | LZD |  | TEC |  |  |
| Non-DDPs | + |  |  |  |  | + |  |  |  |  |  |  |  | 7 |
| Bacteria |  |  |  |  |  |  |  |  |  |  |  |  |  |  |
| *Staphylococcus* | + |  |  |  |  | + |  |  |  | + |  | + |  | 7 |
| *Enterococcus* | + |  |  |  |  | + |  |  |  | + |  | + |  | 7 |
| *Streptococcus* | + |  |  |  |  | + |  |  |  |  |  |  |  | 7 |
| *Acinetobacter baumannii* | + | + |  |  |  | + |  |  |  |  |  |  |  | 14 |
| *Pseudomonas aeruginosa* | + |  | + |  |  | + |  |  |  |  |  |  |  | 14 |
| *Klebsiella pneumoniae* | + |  |  | + |  | + |  |  |  |  |  |  |  | 14 |
| *Escherichia coli* | + |  |  |  |  | + |  |  |  |  |  |  |  | 7 |
| *Enterobacter cloacae* | + |  |  |  |  | + |  |  |  |  |  |  |  | 7 |
| *Haemophilus influenzae* | + |  |  |  |  | + |  |  |  |  |  |  |  | 7 |
| *Burkholderia cepacia* | + |  |  |  |  | + |  |  |  |  |  |  |  | 7 |
| *SMA* | + |  |  |  |  | + |  |  |  |  |  |  |  | 7 |
| *Serratia marcescens* | + |  |  |  |  | + |  |  |  |  |  |  |  | 7 |
| *Others* | + |  |  |  |  | + |  |  |  |  |  |  |  | 7 |
| Fungus |  |  |  |  |  |  |  |  |  |  |  |  |  |  |
| *Candida* | + |  |  |  |  | + |  |  |  |  |  |  |  | 7 |
| *Aspergillus* | + |  |  |  |  |  | + |  |  |  |  |  |  | 14 |
| *Saccharomycetes* | + |  |  |  |  | + |  |  |  |  |  |  |  | 7 |
| *Rhizopus* | + |  |  |  |  |  |  | + |  |  |  |  |  | 7 |
| Virus |  |  |  |  |  |  |  |  |  |  |  |  |  |  |
| *Polyomavirus* | + |  |  |  |  | + |  |  |  |  |  |  |  | 7 |
| *Herpes virus* | + |  |  |  | + | + |  |  |  |  |  |  |  | 7 |
| *Torque teno virus* | + |  |  |  |  | + |  |  |  |  |  |  |  | 7 |
| *Others* | + |  |  |  |  | + |  |  |  |  |  |  |  | 7 |

KT: Kidney transplantation; LT: Liver transplantation; Non-DDPs: Non-donor-derived pathogens; TGC: Tigecycline; POLB: Polymyxin B; CAZ-AVI: Ceftazidime-Avibactam; GCV: Ganciclovir; CAS: Caspofungin; VRC: Voriconazole; ABCD: Amphotericin B cholesterol sulfate complex; LZD: linezolid; TEC: Teicoplanin; *SMA*：*Stenotrophomonas maltophilia*

**Table S2. Univariate analysis of risk factors for donor infection**

TBIL: Total bilirubin; SCR: Serum creatinine; OR: Odds ratios

| Characteristics | Infected donors  (n=270) | Non-infected donors  (n=32) | p-value | OR | 95% confidence interval | |
| --- | --- | --- | --- | --- | --- | --- |
| Age (years) | 44.45 ± 0.88 | 45.41 ± 1.82 | 0.717 | 0.095 | 0.978 | 1.032 |
| Gender, male, n (%) | 194 (71.8%) | 20 (62.5%) | 0.271 | 1.532 | 0.714 | 3.286 |
| Cause of death, n (%) |  |  | 0.255 | 1.000 | 0.848 | 1.179 |
| Trauma | 114 (42.2%) | 16 (50.0%) |  |  |  |  |
| Cerebrovascular accident | 85 (31.5%) | 7 (21.9%) |  |  |  |  |
| Others | 71 (26.3%) | 9 (28.1%) |  |  |  |  |
| TBIL (umol/L) | 23.06 ± 1.17 | 28.24 ± 3.69 | 0.154 | 1.012 | 0.995 | 1.029 |
| SCR (umol/L) | 104.9 ± 4.68 | 117.6 ± 9.52 | 0.364 | 1.002 | 0.998 | 1.006 |
| Endotracheal intubation days | 12.44 ± 0.43 | 8.84 ± 0.89 | **0.006** | **0.904** | **0.885** | **0.924** |
| Length of stay (days) | 13.14 ± 0.49 | 9.43 ± 0.93 | **0.011** | **0.913** | **0.895** | **0.930** |
